# Prevalence, risk factors and treatment outcomes of fluoroquinolones-associated tendinopathy in tuberculosis patients at university hospital, Thailand

**DOI:** 10.1016/j.heliyon.2023.e20331

**Published:** 2023-09-20

**Authors:** Jirarat Chongboonwatana, Varalee Terbsiri, Gompol Suwanpimolkul

**Affiliations:** a Department of Medicine, Faculty of Medicine, Chulalongkorn University, and King Chulalongkorn Memorial Hospital, Thai Red Cross Society, Bangkok, Thailand; b Division of Infectious Diseases, Faculty of Medicine, Chulalongkorn University, and King Chulalongkorn Memorial Hospital, Thai Red Cross Society, Bangkok, Thailand; c Center of Excellence in Tuberculosis, Faculty of Medicine, Chulalongkorn University, Bangkok, Thailand; d Emerging Infectious Diseases Clinical Center, Thai Red Cross Society, Bangkok, Thailand

**Keywords:** Tuberculosis, Drug resistant tuberculosis, Fluoroquinolones, Tendinopathy

## Abstract

**Background:**

Tuberculosis (TB) is an epidemic disease in Thailand. Fluoroquinolones are used to treat TB and have a lengthy treatment course. Therefore, many patients may have adverse effects from these medications. Tendinopathy has been reported as a significant adverse effect of fluoroquinolones. Although the mechanism of tendinopathy from fluoroquinolones is not fully understood, it can progress to a more serious consequence such as a ruptured tendon which can result in morbidity if not treated effectively.

**Methods:**

This study was a single-centered, retrospective descriptive study conducted in patients at a tertiary-level university hospital in Thailand. TB patients who received fluoroquinolones for the treatment of TB from January 2017 to December 2019 were enrolled. This study assessed the prevalence, clinical characteristics, treatment, treatment outcomes for fluoroquinolones-associated tendinopathy, and treatment outcomes of TB.

**Results:**

During the study period, 184 participants that were diagnosed with TB and used fluoroquinolones were enrolled in the study. 34 (18.5%) participants developed tendinopathy. The risk factors that were associated with fluoroquinolones-associated tendinopathy were younger age (<60 years) (Odd ratio (OR) 3.61; 95% CI 1.16–11.23), female (OR 3.54; 95% CI 1.58–7.90), and prolonged usage of levofloxacin (>180 days) (OR 2.61; 95%CI 1.12–6.08). All participants who developed tendinopathy received conservative treatment; the dose of fluoroquinolones was reduced in 9 (26.4%) participants, fluoroquinolones were discontinued in 7 (20.6%) participants and the rest of the participants (n = 18; 52.9%) had conservative treatment. After conservative treatment, 25 (73.5%) participants recovered from tendinopathy. For the TB treatment, 27 (79.4%) participants in the tendinopathy group completed TB treatment and none of them experienced treatment failure. On the other hand, 89 (59.3%) participants in the no tendinopathy group had completed their TB treatment and 3 (2%) of them experienced treatment failure.

**Conclusions:**

The prevalence of fluoroquinolones-associated tendinopathy was not uncommon, and the risk of fluoroquinolones-associated tendinopathy was high in young and female patients. Levofloxacin use was related to an elevated risk of developing tendinopathy, which was dose- and duration-dependent. Conservative treatment, reducing the dose or discontinuation of fluoroquinolones successfully improved the symptoms of tendinopathy. Fluoroquinolones-associated tendinopathy did not affect the treatment of TB.

## 1 Introduction

Tuberculosis (TB) continues to be a serious problem in Thailand even though the Department of Disease Control has already established numerous treatment and prevention guidelines. However, outbreaks of TB were discovered in many areas in Thailand and there were more drug-resistant TB cases found. The guidelines management from World Health Organization (WHO) recommendation 2017 update and the National tuberculosis control programme guidelines in Thailand (NTP) 2018 recommend that the first-line of treatment for drug-susceptible TB is a standard 6-month based regimen composed of isoniazid, rifampicin, pyrazinamide, and ethambutol. As for the drug-resistant TB, WHO consolidated guideline on drug-resistant TB treatment 2018 and NTP 2018 recommended using rifampicin, ethambutol, pyrazinamide and levofloxacin for a duration of 6 months in patients with isoniazid-resistant TB (Hr-TB). Besides this, the treatment for multidrug-resistant TB (MDR-TB), WHO recommended longer MDR-TB regimens which composed of 3 drug groups: Group A (Levofloxacin (Lfx) or moxifloxacin (Mfx), Bedaquiline, and Linezolid), Group B (add one or both of clofazimine and cycloserine or terizidone), and Group C (add to complete the regimen and when medicines from group A and B cannot be used, drugs from group C are Ethambutol, Delenamid, Pyrazinamide, Imipenem-cilastatin, Meropenem, Amikacin or Streptomycin, Ethionamide or prothionamide, and *p*-aminosalicylic acid) for a duration of 18–20 months. In Thailand, NTP 2018 recommended shorter MDR-TB regimens for duration of 9–11 months using kanamycin (Km), moxifloxacin (Mfx), prothionamide (Pto), clofazimine (Cfz), pyrazinamide (Z), high dose isoniazid (H), ethambutol (E), 4–6 Km-Mfx-Pto-Cfz-Z-H (high dose)-E/5Mfx-Cfz-Z-E. According to the guidelines, Fluoroquinolones (FQs) were consisted in many drug-resistant TB regimens. Therefore, fluoroquinolones are increasingly used nowadays. In addition, the duration of TB treatment usually has a protracted treatment period compared to other infections that usually use fluoroquinolones for only a few days. As a result of this, the TB patients are more likely to experience the adverse effects from these drugs. The adverse effect of fluoroquinolones that was disclosed and labeled as a "black box warning" by the US Food and Drug Administration in July 2008 is tendinopathy [1]. If this condition is untreated, it can progress to a more serious condition such as a ruptured tendon which is more harmful and may eventually result in lifelong disability.

The first case of fluoroquinolone-associated tendinopathy was described in 1983 [2,3]. The first case of tendon rupture related to fluoroquinolones was reported in 1991 [4]. The most impacted tendon is the Achilles tendon [3,4]. Although several theories exist, the exact mechanism of fluoroquinolone-induced tendon toxicity remains undefined. In 1998, Williams RJ et al. [5], studied the effects of Fluoroquinolones (Ciprofloxacin) on the tendon, paratenon, and the metabolism of capsular fibroblast. They detected that there was a direct effect of ciprofloxacin on fibroblast metabolism by inhibiting the fibroblast matrix synthesis and fibroblast proliferation, as well as increasing the fibroblast-derived matrix-degrading proteolytic activity in vitro. These observed effects may play a role in the development of tendinitis, tendon degeneration, and tendon rupture associated with the use of fluoroquinolone drugs [5].

To prevent fluoroquinolones-associated tendinopathy, the first step is to identify high-risk individuals and treat them with a different antibiotic whenever possible. When fluoroquinolone is necessary, patients with musculoskeletal problems should be aware of the fluoroquinolone's adverse effect in order to facilitate early recognition, evaluation, and treatment. The patient should limit high-intensity physical activity while on fluoroquinolone therapy or have a history of tendon, joint, or muscle diseases [4]. Concurrent corticosteroid use should be avoided because the steroid may enhance the risk of developing fluoroquinolone-associated tendinopathy [4,6].

The treatment for fluoroquinolone-associated tendinopathy is to discontinue the use of fluoroquinolone if symptoms develop and protect the symptomatic area from additional injury. The patient should gradually return to physical activities based on the symptoms or initiate further diagnostic evaluation and treatment as clinically indicated [4].

There is no data on fluoroquinolone-associated tendinopathy from Southeast Asia. Furthermore, tendinopathy from fluoroquinolones is not fully understood, thus, this study assessed the prevalence, clinical characteristics, risk factors, treatment, and treatment outcome of fluoroquinolones-associated tendinopathy. The findings from this study will provide pertinent information on how to prevent this complication.

## 2 Materials and methods

### 2.1 Study participants

We conducted a single-center, retrospective, descriptive study among patients who received fluoroquinolones for TB treatment at the King Chulalongkorn Memorial Hospital, a tertiary-level university hospital in Thailand, between January 2017 and December 2019. Tendinopathy was defined as joint pain, tendinitis, or tendon rupture. The inclusion criteria were age ≥18 years and were prescribed fluoroquinolone for the treatment of TB. We excluded patients who have fluoroquinolone-resistant TB, tendinitis prior to receiving fluoroquinolone medication, or who have tendinopathy from an accident. The study protocol was approved by the local ethics committee at the Faculty of Medicine, Chulalongkorn University (IRB No.557/63). Written informed consent was waived since this was a retrospective study.

### 2.2 Study design and statistical analysis

We collected data from the outpatient and inpatient medical records of patients diagnosed with TB and treated with fluoroquinolones at the King Chulalongkorn Memorial Hospital, Thai Red Cross Society, between January 1, 2017, and December 31, 2019. The use of medical records had previously been approved by the Director of Chulalongkorn Hospital, Thai Red Cross Society, and for this study, there was no direct interaction with the study participants.

We screened potential eligible participants according to the following International Classification of Diseases, 10th revision (ICD-10) codes: A15.0–19.9 TB with the use of fluoroquinolones. We reviewed each medical record and recorded the following information in a case record form: demographic data (age, sex, weight, body mass index (BMI), underlying disease, etc.), history of TB treatment (organ involvement and drug resistance), administration of fluoroquinolone (levofloxacin, moxifloxacin, or ofloxacin), the fluoroquinolone dose, dates of first and last fluoroquinolone dose, date of onset of symptoms of tendinopathy (if any), clinical characteristics of tendinopathy, treatment outcomes of tendinopathy (fully recovery, partially recovery, treatment failure, tendon rupture, no progress note, lost to follow-up, or death), and treatment outcomes of TB (completed treatment, treatment failure, referred out, lost to follow-up, or death). We also recorded the concurrent use of steroids (prednisolone), immunosuppressive drugs (cyclosporine, tacrolimus, or mTOR inhibitor) and antiproliferative drugs (azathioprine or mycophenolate mofetil), and level of serum creatinine.

Demographic and clinical data were used to describe the participants. Continuous variables were presented as median (interquartile range: IQR). Categorical variables were presented as percentages. Wilcoxon rank-sum test was used to detect the differences in the continuous variables between both groups. The Chi-square test or Fisher exact test was used to detect the differences in the categorical variables between tendinopathy and no tendinopathy groups. Logistic regression was used to determine the factors associated with tendinopathy; covariates from the univariate model with p < 0.1 were adjusted in the multivariate models and stepwise backward logistic regression was used to select the final model. All P-values reported were two-sided. Statistical significance was defined as P < 0.05. Stata version 15.1 (Stata Corp., College Station, Texas) was used for statistical analysis.

### 2.3 Primary and secondary endpoints

The primary endpoint was to determine the prevalence of fluoroquinolones-associated tendinopathy in TB patients during the study period. The secondary endpoints assessed the risk factors that were associated with fluoroquinolones-associated tendinopathy, the treatment and treatment response for patients with fluoroquinolones-associated tendinopathy, and the treatment outcome of TB.

## 3 Results

A total of 2429 patients diagnosed with TB between January 2017 and December 2019 were screened. 184 patients were diagnosed with TB and were prescribed fluoroquinolones. These patients were enrolled into the study. 68 (37%) participants were female with a mean age of 49 (IQR 36–63) years (Table 1).

**Table 1:** Baseline characteristics of the study participants.

Characteristics, n (%)	Total (N = 184)	No Tendinopathy (N = 150)	Tendinopathy (N = 34)	P-value
Age (years), median (IQR)	49 (36–63)	49 (36–66)	47 (38–55)	0.46
Age groups, n (%)				0.02
<60	131 (71.2)	101 (67.3)	30 (88.2)	
≥60	53 (28.8)	49 (32.7)	4 (11.8)	
Female, n (%)	68 (37)	48 (32)	20 (58.8)	0.003
BMI (kg/m^2^), median (IQR)	20.8 (18–23.4)	20.7 (17.8–23.4)	21.09 (18.95–23.65)	0.32
BMI (kg/m^2^), n (%)				0.18
<23	110 (59.8)	88 (58.7)	22 (64.7)	
≥23	44 (23.9)	34 (22.7)	10 (29.4)	
Unavailable data	30 (16.3)	28 (18.7)	2 (5.9)	
Underlying disease/condition, n (%)				
Diabetes mellitus	39 (21.2)	34 (22.7)	5 (14.7)	0.31
Hypertension	37 (20.1)	32 (21.3)	5 (14.7)	0.38
Dyslipidemia	24 (13)	22 (14.7)	2 (5.9)	0.17
HIV	37 (20.1)	33 (22)	4 (11.8)	0.18
Gout	1 (0.5)	0 (0)	1 (2.9)	0.04
Heart transplant	4 (2.2)	2 (1.3)	2 (5.9)	0.16
Liver transplant	2 (1.1)	2 (1.3)	0 (0)	0.50
Kidney transplant	5 (2.7)	5 (3.3)	0 (0)	0.59
Stem cell transplant	2 (1.1)	2 (1.3)	0 (0)	0.50
Creatinine (mg/dL), median (IQR)	0.81 (0.64–0.95)	0.84 (0.65–0.96)	0.75 (0.62–0.9)	0.19
eGFR (min/mL/1.73m^2^), median (IQR)	99.9 (76.9–114.6)	99.4 (74.7–114.5)	100.3 (94.5–115.2)	0.40
eGFR <60, n (%)	25 (13.6)	23 (15.3)	2 (5.9)	0.18
Creatinine clearance, median (IQR)	80.9 (54.1–109.4)	77.9 (53–111.7)	82.3 (72.4–105.2)	0.28
Creatinine clearance <60^a^ (mL/min), n (%)	53 (28.8)	50 (33.3)	3 (8.8)	0.004
CKD stage ≥3, n (%)	9 (4.9)	8 (5.3)	1 (2.9)	0.60

a Cockcroft-Gault creatinine clearance (mL/min).

Out of 184 participants that were diagnosed with TB and treated with fluoroquinolones, 34 (18.5%) participants experienced tendinopathy as an adverse effect of fluoroquinolone, while 150 (81.5%) participants did not develop tendinopathy during the study period. Among the participants that developed tendinopathy, most of them were female (58.8%, p 0.003) and the mean age was 47 (IQR 38–55) years. The tendinopathy frequently occurred in participants that were younger than 60 years old (88.2% and 11.8% in the age group of <60 and ≥ 60 years, respectively, p 0.02). The participants that had gout as an underlying disease also significantly experienced tendinopathy (2.9%, p 0.04). Body weight, renal function, and other underlying diseases were not associated with the development of tendinopathy (Table 1).

There was no statistically significant difference in the organ involvement of TB between those who developed tendinopathy and those who did not. Participants diagnosed with drug-resistant TB, isoniazid-resistant TB patients (52.9%, p 0.04) were more likely to have tendinopathy than those with other drugs resistant to TB. The fluoroquinolone that was mostly used in the participants who developed tendinopathy was levofloxacin (94.1%, p 0.08) and the most often prescribed dose was 750 mg/day (58.5%). The median duration of levofloxacin use was 354.5 days (IQR 171–508). The second fluoroquinolones used was ofloxacin. 5 participants (14.7%, p 0.11) were on ofloxacin, and the prescribed dose was usually 600–799 mg/day; the median duration of ofloxacin use was 46 days (IQR 15–48) (Table 2). The rationale for using fluoroquinolones for TB treatment in this study were adverse effects of other anti-TB drugs, drug resistance to the first-line of treatment, drug-drug interactions, and clinically not responsive to TB treatment. Of 184 participants, 42.2% of the participants had adverse effects from other anti-TB medications. 39.1% of the participants had drug resistance to the first-line treatment. 13% of the participants had drug-drug interaction. 5.4% of the participants did not clinically respond to the first-line treatment (Supplemental Table 1). Of the concomitant drugs used with TB treatment, 13.6% of the participants were on steroids and 8.2% of the participants were on immunosuppressive drugs; there were no significant differences in the development of fluoroquinolones-associated tendinopathy among these participants (Supplemental Table 2).

**Table 2:** Tuberculosis organ involvement, drug resistant information and treatment of tuberculosis.

	Total (N = 184)	No Tendinopathy (N = 150)	Tendinopathy (N = 34)	P-value
Tuberculosis organ involvement, n (%)				
Pulmonary	146 (79.4)	119 (79.3)	27 (79.4)	0.99
Extrapulmonary^a^	38 (20.6)	31 (20.7)	7 (20.6)	0.99
Pleura	10 (5.4)	8 (5.3)	2 (5.9)	0.90
Pericardium	2 (1.1)	2 (1.3)	0 (0)	0.50
Peritoneum	4 (2.2)	4 (2.7)	0 (0)	0.34
Central nervous system	9 (4.9)	8 (5.3)	1 (2.9)	0.60
Lymph node	32 (17.4)	27 (18)	5 (14.7)	0.65
Musculoskeletal system	16 (8.7)	13 (8.7)	3 (8.8)	0.98
Genitourinary system	6 (3.3)	5 (3.3)	1 (2.9)	0.91
Gastrointestinal system	9 (4.9)	6 (4)	3 (8.8)	0.37
Mucocutaneous system	2 (1.1)	2 (1.3)	0 (0)	0.50
Hematologic organs	13 (7.1)	10 (6.7)	3 (8.8)	0.71
Endocrine organs	1 (0.5)	1 (0.7)	0 (0)	0.63
Ocular	2 (1.1)	2 (1.3)	0 (0)	0.50
Disseminated	37 (20.1)	31 (20.7)	6 (17.7)	0.69
TB drug resistance, n (%)				
● Isoniazid	69 (37.5)	51 (34)	18 (52.9)	0.04
● Rifampicin	30 (16.3)	24 (16)	6 (17.7)	0.81
● Pyrazinamide	6 (3.3)	4 (2.7)	2 (5.9)	0.31
● Ethambutol	4 (2.2)	3 (2)	1 (2.9)	0.56
● Streptomycin	21 (11.4)	18 (12)	3 (8.8)	0.77
Levofloxacin, n (%)	152 (82.6)	120 (80)	32 (94.1)	0.08
● Dose (mg/day), n (%)				0.13
<500	6 (3.3)	6 (4)	0 (0)	
500	52 (28.3)	40 (26.7)	12 (35.3)	
750	94 (51.1)	74 (49.3)	20 (58.8)	
● Exposure duration (days), median (IQR)	261 (115.5–409.5)	231 (107–388.5)	354.5 (171–508)	0.12
Moxifloxacin, n (%)	4 (2.2)	4 (2.7)	0 (0)	0.34
Ofloxacin, n (%)	47 (25.5)	42 (28)	5 (14.7)	0.11
● Dose (mg/day), n (%)				0.44
400-599	9 (4.9)	9 (6)	0 (0)	
600-799	22 (12)	19 (12.7)	3 (8.8)	
≥800	16 (8.7)	14 (9.3)	2 (5.9)	
● Exposure duration (days), median (IQR)	80 (16–392)	90 (20–392)	46 (15–48)	0.60

a *Extrapulmonary tuberculosis* is defined as no pulmonary involvement, disseminated tuberculosis with pulmonary and other extrapulmonary involvement not included.

### 3.1 Risk factors associated with tendinopathy

Logistic regression analysis was used to identify the risk factors for developing tendinopathy in TB patients who used fluoroquinolones. The risk factors for developing tendinopathy were age, sex, and the use of levofloxacin. Participants younger than 60 years old had a significantly higher risk for developing tendinopathy after using fluoroquinolones (univariate analysis: OR 3.63; 95% CI 1.21–10.91; p 0.02 and multivariate analysis: OR 3.61 95% CI 1.16–11.23; p 0.03). Female participants were more likely to develop tendinopathy (univariate analysis: OR 3.04; 95% CI 1.41–6.52; p 0.004 and multivariate analysis: OR 3.54; 95% CI 1.58–7.90; p 0.002). Participants who received more than 500 mg/day of levofloxacin were also more susceptible to developing tendinopathy (univariate analysis: OR 5.05; 95% CI 1.12–22.1; p 0.03). Prolonged usage of levofloxacin for more than 180 days could increase the risk of developing tendinopathy (univariate analysis: OR 2.53; 95% CI 1.13–5.65; p 0.02, and multivariate analysis: 2.61; 95% CI1.12-6.08; p 0.03). Additionally, body mass index (BMI), heart transplantation, and the use of cyclosporine were related to the incidence of tendinopathy, but these associations were not statistically significant. (Table 3).

**Table 3:** Risk factors associated with tendinopathy.

	Univariate		Multivariate	
	OR (95%CI)	P-value	aOR (95%CI)	P-value
Age <60 years	3.63 (1.21–10.91)	0.02	3.61 (1.16–11.23)	0.03
Female	3.04 (1.41–6.52)	0.004	3.54 (1.58–7.90)	0.002
BMI (kg/m^2^)	1.07 (0.97–1.18)	0.18		
Underlying				
Diabetes mellitus	0.59 (0.21–1.63)	0.31		
Hypertension	0.64 (0.23–1.77)	0.39		
Dyslipidemia	0.36 (0.08–1.63)	0.19		
HIV	0.47 (0.16–1.44)	0.19		
Heart transplant	4.63 (0.63–34.07)	0.13		
eGFR <60 (ml/min/1.73m^2^)	0.35 (0.08–1.54)	0.16		
Creatinine clearance <60 (mL/min)	0.19 (0.06–0.66)	0.01		
CKD stage ≥3	0.55 (0.07–4.45)	0.57		
Fluoroquinolones				
Levofloxacin used	4 (0.91–17.63)	0.07		
Levofloxacin dose (mg/day)				
500	3.91 (0.82–18.73)	0.09		
750	4.05 (0.89–18.43)	0.07		
Levofloxacin dose >500 mg/day	5.05 (1.12–22.1)	0.03		
Duration of Levofloxacin >180 days	2.53 (1.13–5.65)	0.02	2.61 (1.12–6.08)	0.03
Ofloxacin used	0.44 (0.16–1.22)	0.12		
Ofloxacin Dose (mg/day)				
600-799	0.59 (0.16–2.13)	0.42		
≥800	0.53 (0.11–2.47)	0.42		
Concomitant drugs used				
Prednisolone	0.35 (0.08–1.54)	0.16		
Immunosuppressive drugs	0.66 (0.14–3.07)	0.60		
● Cyclosporine	1.11 (0.12–10.22)	0.93		
● Tacrolimus	0.62 (0.07–5.21)	0.66		
Antiproliferative drugs				
● mTOR inhibitor	0.88 (0.1–7.77)	0.91		
● Mycophenolate mofetil	0.98 (0.2–4.75)	0.98		

OR = Odds ratio, aOR = adjusted Odds ratio. Univariate and Multivariate models were used. Multivariate models were developed by adjusting the covariates with p < 0.1 obtained from the univariate model. Stepwise backward LR was used to select the final model.

### 3.2 Management of fluoroquinolones-associated tendinopathy

All participants who developed tendinopathy as a side effect of fluoroquinolones received conservative (non-surgery) treatment: 9 (26.4%) participants had their fluoroquinolone dose reduced, 7 (20.5%) participants had to discontinue fluoroquinolone, and the rest of the participants (n = 18; 52.9%) had conservative treatment based on observation alone. The average duration of treatment was 74 days (IQR 28.0–146), whereas the average duration of symptoms was 77 days (IQR 41–142). After conservative treatment, 12 (35.3%) participants completely recovered, 13 (38.2%) participants partially recovered, and 2 (5.9%) participants had persistent symptoms and did not recover from tendinopathy. Only one participant was lost to follow-up. During the study period, none of the participants experienced a ruptured tendon or died as a result of fluoroquinolone-associated tendinopathy consequence (Table 4).

**Table 4:** Management and outcome of fluoroquinolones-associated tendinopathy.

Treatment outcome, n (%)	Tendinopathy (n = 34)
Treatment, n (%)	
Conservative	34 (100)
Dose reduction	9 (26.5)
Levofloxacin	7 (77.8)
Moxifloxacin	0 (0)
Ofloxacin	2 (22.2)
Discontinuation of fluoroquinolone	7 (20.6)
Levofloxacin	6 (85.7)
Moxifloxacin	0 (0)
Ofloxacin	1 (14.2)
Observation	18 (52.9)
Levofloxacin	18 (100)
Moxifloxacin	0 (0)
Ofloxacin	0 (0)
Surgery	0 (0)
Duration of treatment (days), median (IQR)	74 (28–146)
Duration of symptoms (days), median (IQR)	77 (41–142)
Tendinopathy treatment outcome, n (%)	
Fully recovery	12 (35.3)
Partially recovery	13 (38.2)
Treatment failure^a^	2 (5.9)
Tendon rupture	0 (0)
No progress note	6 (17.6)
Lost to follow-up	1 (2.9)
Death	0 (0)

a No clinical improvement.

### 3.3 Tuberculosis treatment outcome

The result of TB treatment in 184 participants that were diagnosed with TB and treated with fluoroquinolones, 116 (63.0%) participants had completed treatment courses for TB; 44 (23.9%) participants were lost to follow-up before completing the treatment for TB, 9 (4.9%) participants were referred out to other hospitals based on their health care providers, 3 (1.6%) participants had treatment failure and 12 (6.5%) participants died. The cause of death in 12 participants was not related to the side effects of fluoroquinolones. In the tendinopathy group, 27 (79.4%) participants completed TB treatment and none of the participants experienced treatment failure. On the other hand, there were 89 (59.3%) participants who completed the TB treatment in the no tendinopathy group, and 3 (2%) participants in this group had experienced treatment failure.

More specifically by dividing the TB treatment outcome into three groups based on drug susceptibility: 1) drug-susceptible, 2) isoniazid-resistant (Hr-TB), and 3) multidrug-resistant or rifampicin-resistant (MDR/RR-TB). In the drug-susceptible TB patients with tendinopathy, 12 (75.0%) participants completed TB treatment. Out of these 12 participants that completed TB treatment, 3 participants had reduced their fluoroquinolones dose, 2 participants discontinued fluoroquinolones and 7 participants continued their TB treatment based on clinical observation alone. None of these participants in drug-susceptible patients with tendinopathy group experienced treatment failure. For drug-susceptible TB patients with no tendinopathy, 52 (55.9%) participants completed TB treatment and 2 (2.1%) participants experienced treatment failure. In Hr-TB patients with tendinopathy, 9 (90.0%) participants completed TB treatment, 3 of them had reduced their fluoroquinolones dose, 3 of them discontinued fluoroquinolones, and 3 of them continued their TB treatment with clinical observation alone, and none of the participants experienced treatment failure. For Hr-TB patients with no tendinopathy, 12 (54.5%) participants completed TB treatment and 1 (4.6%) participant in this group experienced treatment failure. In MDR/RR-TB patients with tendinopathy, 6 (75.0%) participants completed TB treatment, 1 of them had reduced the dose of fluoroquinolones, 1 of them discontinued fluoroquinolones, and 4 of them continued their TB treatment with clinical observation alone, and none of the participants experienced treatment failure. For MDR/RR-TB patients with no tendinopathy, 25 (71.4%) participants completed TB treatment and none of the participants experienced treatment failure (Table 5).

**Table 5:** Tuberculosis treatment outcome.

Treatment outcome, n (%)	Total (N = 184)	No tendinopathy (n = 150)	Tendinopathy (n = 34)
All cases			
Completed treatment	116 (63.0)	89 (59.3)	27 (79.4)
Treatment failure	3 (1.6)	3 (2.0)	0 (0)
Referred out	9 (4.9)	7 (4.7)	2 (5.9)
Lost to follow-up	44 (23.9)	39 (26.0)	5 (14.7)
Death	12 (6.5)	12 (8.0)	0 (0)
**Drug-susceptible group**	**109 (59.2)**	**93 (62.0)**	**16 (47.0)**
Completed treatment	64 (58.7)	52 (55.9)	12 (75.0)
FQs dose reduction			3
Discontinue FQs			2
Observation			7
Treatment failure	2 (1.8)	2 (2.1)	0 (0)
Referred out	4 (3.7)	3 (3.2)	1 (6.25)
Lost to follow-up	27 (24.8)	24 (25.8)	3 (18.75)
Death	12 (11.5)	12 (12.9)	0 (0)
**Hr-TB**	**32 (17.4)**	**22 (14.7)**	**10 (29.4)**
Completed treatment	21 (65.6)	12 (54.5)	9 (90.0)
FQs dose reduction			3
Discontinue FQs			3
Observation			3
Treatment failure	1 (3.13)	1 (4.6)	0 (0)
Referred out	1 (3.13)	1 (4.6)	0 (0)
Lost to follow-up	9 (28.1)	8 (36.4)	1 (10.0)
Death	0 (0)	0 (0)	0 (0)
**MDR/RR-TB**	**43 (23.4)**	**35 (23.3)**	**8 (23.5)**
Completed treatment	31 (72.1)	25 (71.4)	6 (75.0)
FQs dose reduction			1
Discontinue FQs			1
Observation			4
Treatment failure	0 (0)	0 (0)	0 (0)
Referred out	4 (9.3)	3 (8.5)	1 (12.5)
Lost to follow-up	8 (18.6)	7 (20.0)	1 (12.5)
Death	0 (0)	0 (0)	0 (0)

FQs = fluoroquinolones.

Hr-TB = Isoniazid-resistant tuberculosis.

MDR/RR-TB = Multidrug- or rifampicin-resistant tuberculosis.

## 4 Discussion

Several studies in the past three decades have revealed the incidence of fluoroquinolones-associated tendinopathy. In 1994, a nested case-control study conducted in Belgium discovered that the incidence of Achilles tendinopathy in renal transplant patients treated with fluoroquinolones was 12.2% (p < 0.02) [7]. In 1999, a retrospective cohort study conducted in the Netherlands assessed the association between fluoroquinolone use and tendinitis; the incidence rate of tendinitis during the use of fluoroquinolones in this study was 7.74 per 100,000 days at risk [8]; and in 2008, an observational cohort study from Spain found that an individual risk incidence of developing Achilles tendinopathy in heart transplant patients was 5.8% (95% CI 2.8 to 8.7) [9] In our study, the prevalence of tendinopathy was 18.5% in the participants who received fluoroquinolones for long-term TB treatment. Even though the findings were consistent with previous reports and verified that the probability of a patient developing tendinopathy as a result of fluoroquinolone treatment was not unusual. However, the prevalence of fluoroquinolone-associated tendinopathy in our study was substantially higher. This discrepancy may be due to the duration of TB treatment using fluoroquinolones. Long-term use of fluoroquinolones in our study can increase the risk of developing side effects. And, to clarify other causes that might increase the prevalence, pyrazinamide can also cause joint pain. However, this side effect usually appears during the first one or two months of treatment [10]. In our study, most of the participants in the tendinopathy group did not use pyrazinamide at the time when the symptoms occurred, and the rest of the participants used pyrazinamide for more than two months before the symptoms occurred.

A previous study by van der Linden et al. revealed that the risk factor for developing Achilles tendinitis was higher among the elderlies [6]. In our study, fluoroquinolone-associated tendinopathy was frequently diagnosed in participants younger than 60 years old compared to previous studies. This can be explained that our participants were mostly younger, and the sample size was smaller compared to the previous studies. In addition, older patients in Asia are usually endurable and do not report pain to physicians compared to the younger generation. Therefore, the prevalence of fluoroquinolone-associated tendinopathy was higher among younger patients. We also found that the tendinopathy occurred more in female participants, and the use of steroids or immunosuppressive drugs did not increase the risk of developing fluoroquinolone-associated tendinopathy. In contrast to the previous study by Lewis et al. and Barge-Caballero et al., patients treated with fluoroquinolones had a higher risk of developing Achilles tendon disorders; the risk factors for developing Achilles tendinitis were higher among male patients and concomitant use of steroids [3,9]. The results from these previous studies were incomparable to our study because our study had a longer observational period to assess the effects of fluoroquinolone-associated tendinopathy.

Numerous previous reports indicated that levofloxacin is more likely to cause tendon injury compared to the other fluoroquinolones, and the risk appears to be exposure dependent; higher doses and longer durations of TB treatment were most commonly associated with tendinopathy [11]. Likewise, our study discovered that levofloxacin use increased the risk of developing tendinopathy. Levofloxacin doses greater than 500 mg/day and durations longer than 180 days increased the risk of developing tendinopathy.

### 4.1 Treatment and treatment outcomes of fluoroquinolone-associated tendinopathy

In a previous study, there was a good prognosis after the withdrawal of fluoroquinolones, with more than 80% of the patients achieving complete recovery within two months [9,12]. In our study, 73.5% of the participants recovered when conservative treatment was used. The conservative treatment in our study, defined as non-surgery treatment included a reduced dosage of fluoroquinolones (26.5%), discontinuation of fluoroquinolone treatment (20.6%), or observation alone (52.9%). The fluoroquinolone that was most commonly affected was levofloxacin. The average duration of the symptoms in our study was 77 days. Thus, the results of our study and previous study were correlated and pointed toward a good prognosis after conservative treatment for fluoroquinolone-associated tendinopathy.

### 4.2 Treatment and treatment outcomes of tuberculosis

In the treatment guidelines, fluoroquinolones are recommended for use among patients with drug-resistant TB. Our study found that there were other reasons to change treatment from first-line drugs to fluoroquinolones. The first reason to change the treatment from the first-line drugs to fluoroquinolones was the adverse effects of other anti-TB drugs (42.2%). For example, drug-induced liver injury from isoniazid, rifampicin, or pyrazinamide, and optic neuritis from ethambutol. Second, first-line therapy was changed to fluoroquinolones due to drug-resistant TB (39.1%). Third, drug-drug interaction (13.0%), especially with antiviral drugs for HIV patients and immunosuppressive drugs for transplant patients, are another reason for changing first-line therapy to fluoroquinolones. Last, was the use of fluoroquinolones when the patients were clinically not responsive after receiving the first-line TB treatment (5.4%) (Supplemental Table 1).

After the patients experienced fluoroquinolone-associated tendinopathy, the problem that many physicians had to confront was whether the fluoroquinolone-associated tendinopathy itself nor the conservative treatment of fluoroquinolone-associated tendinopathy by reducing the dosage of fluoroquinolone or discontinuing the use of fluoroquinolone might affect the TB treatment outcomes. Our study found that even though the patients experienced fluoroquinolone-associated tendinopathy and had decreased fluoroquinolones or discontinued the use of fluoroquinolones, there were 79.4% of the participants who completed their TB treatment and there was no treatment failure and adverse effects reported in the tendinopathy group. In addition, when the TB treatment outcomes of the tendinopathy group were compared to the no tendinopathy group, the TB treatment outcomes in the tendinopathy group were better than the no tendinopathy group (79.4%, 59.3%, respectively). The treatment failure patients were reported only in the no tendinopathy group (2%). Furthermore, even when dividing TB treatment outcomes into subgroups based on drug susceptibility (drug-susceptible TB, Hr-TB, and MDR/RR-TB), the results between all three subgroups were not different. Aside from that, the patients that were lost to follow-up, referred to another hospital or died from other causes that were not associated with fluoroquinolone treatment.

As far as we know, there was no study that assessed the prevalence and risk factors for fluoroquinolones-associated tendinopathy among TB patients in a Southeast Asian country. Previous studies were conducted in other regions of the world where there are different ethnicities, weights, and genetic susceptibility to develop tendinopathy. Aside from that, the duration of fluoroquinolone treatment in the previous study was concise compared to our study. The participants in our study had longer periods of fluoroquinolone treatment, which is why we were able to show a greater association between fluoroquinolone and tendinopathy and can also reflect TB treatment outcomes in the patient that had fluoroquinolones-associated tendinopathy from TB treatment.

There were some limitations in this study. First, at the time of our investigation, the hospital had transitioned from paper medical records to electronic medical records. Consequently, some information and data may not be available. Second, there was a knowledge gap regarding fluoroquinolone's adverse effects in Thailand. As a result, many physicians were unable to notice and report this adverse event adequately. Third, the sample size was small. Last, the study design may contribute to selection bias. This was a retrospective, non-randomization study. We recommend that further randomized control trials with a larger sample size be conducted to determine the risk of fluoroquinolones-associated tendinopathy in TB patients.

In conclusion, according to our knowledge, this is the first study of fluoroquinolones-associated tendinopathy conducted in TB patients in Southeast Asia. The results of this study showed that the prevalence of fluoroquinolones-associated tendinopathy among TB patients was not uncommon. The risk factors that contribute to the development of fluoroquinolones-associated tendinopathy were significantly higher among younger (<60 years) and female patients. Fluoroquinolones, particularly levofloxacin, were significantly associated with an increased risk of developing tendinopathy. The development of fluoroquinolones-associated tendinopathy was dose-dependent and duration-dependent. Conservative treatment for fluoroquinolones-associated tendinopathy such as reducing the dose of fluoroquinolones or discontinuation of fluoroquinolones can successfully improve the symptoms of tendinopathy. Finally, fluoroquinolone-associated tendinopathy and the treatment of tendinopathy did not affect the treatment of TB.

## Author contribution statement

Jirarat Chongboonwatana: Conceived and designed the experiments; Performed the experiments; Analyzed and interpreted the data; Wrote the paper.

Varalee Terbsiri: Conceived and designed the experiments; Performed the experiments; Analyzed and interpreted the data; Wrote the paper.

Gompol Suwanpimolkul: Conceived and designed the experiments; Performed the experiments; Analyzed and interpreted the data; Contributed reagents, materials, analysis tools or data; Wrote the paper.

## Data availability statement

Data included in article/supplementary material/referenced in article.

## Declaration of competing interest

The authors declare that they have no known competing financial interests or personal relationships that could have appeared to influence the work reported in this paper.
