# Supplementary material for: Prevalence, risk factors and treatment outcomes of fluoroquinolones-associated tendinopathy in tuberculosis patients at university hospital, Thailand
Source: Heliyon. 2023 Sep 20;9(10):e20331. doi: 10.1016/j.heliyon.2023.e20331 (PMC10550594; doi:10.1016/j.heliyon.2023.e20331)
Supplement: Multimedia component 2 [file mmc2.docx]

**Supplemental Table 1** Reasons for using fluoroquinolones for the treatment of tuberculosis.

| **Cause, n(%)** | **Total**  **(N=184)** | **No tendinopathy**  **(n=150)** | **Tendinopathy**  **(n=34)** | **P-value** |
| --- | --- | --- | --- | --- |
| Drug resistance | 72 (39.1) | 55 (36.7) | 17 (50.0) | 0.029 |
| Drug-drug interaction | 24 (13.0) | 21 (14.0) | 3 (8.8) |  |
| Adverse effects due to other anti-tuberculosis drugs | 78 (42.2) | 67 (44.7) | 11 (32.4) |  |
| Clinically not responsive after receiving the first-line treatment | 10 (5.4) | 7 (4.7) | 3 (8.8) |  |

**Supplemental Table 2** Concomitant drug(s) used.

| **Drugs, n(%)** | **Total**  **(N=184)** | **No Tendinopathy**  **(N=150)** | **Tendinopathy**  **(N=34)** | **P-value** |
| --- | --- | --- | --- | --- |
| Prednisolone | 25 (13.6) | 23 (15.3) | 2 (5.9) | 0.18 |
| Immunosuppressive drugs | 15 (8.2) | 13 (8.7) | 2 (5.9) | 0.18 |
| - Cyclosporine | 5 (2.7) | 4 (2.7) | 1 (2.9) | 0.92 |
| - Tacrolimus | 8 (4.4) | 7 (4.7) | 1 (2.9) | 0.66 |
| - mTOR inhibitor | 6 (3.3) | 5 (3.3) | 1 (2.9) | 0.91 |
| Antiproliferative drugs |  |  |  |  |
| - Mycophenolate mofetil | 11 (6.0) | 9 (6.0) | 2 (5.9) | 0.98 |
| - Azathioprine | 1 (0.5) | 1 (0.7) | 0 (0) | 0.63 |
